# Speech-touch integration for affective human–robot interaction: a scoping review

**DOI:** 10.3389/frobt.2026.1785039

**Published:** 2026-05-08

**Authors:** Alastair Howcroft, Maria Elena Giannaccini, Steve Benford, Ahmad Khan, Holly Blake

**Affiliations:** 1 School of Computer Science, University of Nottingham, Jubilee Campus, Nottingham, United Kingdom; 2 Leicester Medical School, University of Leicester, George Davies Centre, Leicester, United Kingdom; 3 School of Health Sciences, University of Nottingham, Queen’s Medical Centre, Nottingham, United Kingdom; 4 NIHR Nottingham Biomedical Research Centre, Nottingham University Hospitals NHS Trust, Nottingham, United Kingdom

**Keywords:** affective touch, artificial intelligence, care robots, empathy, healthcare social robots, scoping review, social robotics

## Abstract

**Background:**

Artificial intelligence is increasingly capable of expressing empathy through language, yet the integration of physical touch–an important cue for social connection–remains fragmented. Although robots utilise language or touch individually, few systems coordinate both modalities, potentially limiting their capacity for affective human-robot interaction (HRI). This scoping review maps social robots that combine spoken language and tactile interaction (e.g., hugging, stroking, warmth, vibration), examines how these modalities are coordinated in existing systems, and synthesises reported user outcomes and design implications.

**Methods:**

Following the Preferred Reporting Items for Systematic Reviews and Meta-Analyses extension for Scoping Reviews (PRISMA-ScR) guidelines, searches across five databases (IEEE Xplore, PubMed, ACM, Web of Science, Scopus) and supplementary web sources identified 11 distinct HRI implementations that pair speech with active or invited touch. Of these, eight implementations included explicit comparison conditions (e.g., speech-only vs. speech + touch, or touch-only vs. touch + speech), enabling assessment of the added value of combining modalities.

**Results:**

Across comparative studies, combining speech and touch showed potential to be more effective than speech-only or touch-only HRI in some contexts. This integration can make robots appear more caring, empathic, and human-like, while strengthening attachment, increasing willingness to self-disclose, and helping users feel calmer (e.g., lower heart rate). However, outcomes were implementation-dependent, with some studies reporting no additional benefit from the combined modalities. Across the evidence base, the review found a consistent suggestive pattern that warm (e.g., near skin temperature), soft, naturalistic touch tends to support more positive affective HRI outcomes than cold, rigid, “mechanical” touch. The evidence base was also largely dominated by short, lab-based studies using existing, typically rigid robotic platforms not purpose-built for affective speech–touch interaction.

**Conclusion:**

Speech–touch integration in social HRI is a small but promising area, particularly for healthcare and emotional-support applications (e.g., supporting children in hospital). Despite this potential, very few robots are purpose-built for coordinated speech and touch. Affective speech–touch HRI remains challenging because of its psychological, socio-cultural, and engineering demands. Progress will likely require soft, safe, warm, and increasingly autonomous systems that move beyond repurposed rigid platforms.

**Systematic Review Registration:**

https://doi.org/10.17605/OSF.IO/2PA6J, identifier OSF.IO/2PA6J.

## 1 Introduction

### 1.1 Language-based empathy in artificial agents

Artificial Intelligence (AI) systems are increasingly able to recognise emotional cues and produce replies perceived as empathic. Across 15 comparative healthcare communication studies, blinded evaluators rated generative AI replies as more empathic than human practitioners’ in 13 (87%) of them, with AI also more likely to be judged the more empathic response in direct, side-by-side text comparisons (about 73% of the time) (Howcroft et al., 2025). Such perceived empathy can improve wellbeing, particularly in health and care settings (MacFarlane et al., 2017; Decety, 2020), and empathic expressions in AI responses can increase perceived supportiveness (Liu and Sundar, 2018). Notably, even simpler designs–where empathic messages are pre-written and delivered by a scripted chatbot in response to user inputs–have been shown to yield small, short-term improvements in mood (De Gennaro et al., 2020). Beyond immediate support, ongoing AI-mediated dialogue can help a sense of connection grow over time, with people sometimes treating the agent a bit like a person or opening up emotionally (Skjuve et al., 2022). These findings suggest that language alone can, in some settings, produce interactions experienced as empathic, even when the reply is not produced by a human. However, to convey empathy in ways that feel more vivid and relational, and to foster a stronger sense of connection between user and system, AI may benefit from moving beyond interaction confined to the screen to include embodied presence (Kwak et al., 2013).

### 1.2 The importance of non-verbal cues and touch

Non-verbal cues from an empathic responder–such as gaze and shifts in tone of voice–are important for conveying empathy and affective meaning, enabling the recipient to perceive the response as more empathic (Kraft-Todd et al., 2017; Rahmanti et al., 2025). Touch may be one of the most direct and powerful of these cues (Della Longa et al., 2021; Buono et al., 2025). Nurses in one qualitative study reported that gentle touch (such as holding a hand) can be an invitation for patients to talk, and sometimes seemed to help them express emotion (Sandnes and Uhrenfeldt, 2024). Experimental work even suggests that touch can convey specific emotions. In lab studies, participants could recognise emotions such as ‘love’, ‘gratitude’, and ‘sympathy’ from brief touches to the arm, and different touch patterns (e.g., stroking vs. patting) were linked to conveying different emotions (Hertenstein et al., 2006). Other experimental work suggests that mediated tactile feedback may convey greater perceived social support and prosocial intent than visual-only digital cues (Saramandi et al., 2024).

Care and therapeutic robots are becoming more common in hospitals and other health and wellbeing settings (Morgan et al., 2022). As AI becomes physically present through social robots, the capacity for touch–being interactive and touchable, like a human–will be increasingly important for expressing social and interpersonal connection (Kelly et al., 2020; Kalinowska et al., 2023; Buono et al., 2025).

Tactile interaction has also been linked to measurable physiological responses. For example, interactions with a soft, tactile companion robot (LOVOT) have been associated with reduced cortisol–a hormone associated with stress–and long-term owners exhibit higher baseline oxytocin, a hormone linked to bonding (Imamura et al., 2023).

However, touch in health and care settings (Buono et al., 2025), as well as physical contact in human–robot interaction (Benford et al., 2025), may convey affective meaning in ways that depend heavily on context, consent, and relationships. When appropriately timed and framed, it may be able to amplify positive emotion and enhance perceived support beyond verbal communication alone (Sawabe et al., 2022). Yet this promise does not make touch easy to translate into robot design.

### 1.3 Limitations of existing systems

Despite the potential of touch as an affective resource, combining speech and touch within the same robot used for social human–robot interaction (HRI) appears uncommon, with only a small number of reported systems coordinating both modalities to express affect and social connection–for example, “Huggable” (Jeong et al., 2015; Figure 3D) and “EmoPus” (Li et al., 2024; Figure 3E) (see Table 1 in the Results section for further implementation details). This limited integration of speech and touch may limit the capacity of such robots for responsive, empathic interaction, because many systems still prioritise one modality over the other.

**FIGURE 1:**
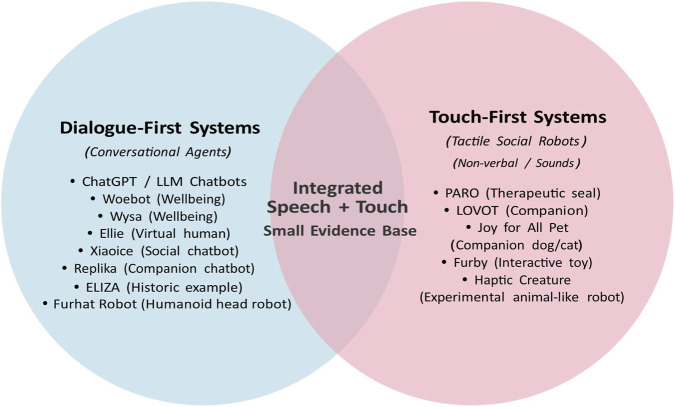
Conceptual framing of two established strands–dialogue-first conversational agents and touch-first tactile companion robots–and the relatively under-studied intersection of systems that combine spoken language with touch for affective use. Examples are illustrative, not exhaustive.

**TABLE 1:** Summary of studies examining social robots that integrate speech and touch for affective human–robot interaction (F = female; M = male; NB = non-binary; NR = not reported).

Implementation	Robot	Dialogue component	Touch component	Purpose of combination	Key results	Authors’ interpretation	Evidence context (sample/setting)
Arnold and Scheutz (2018)	PR2 (teleoperated, no touch sensors)	Scripted verbal dialogue with *positive* (“That’s OK, I know how to fix this”) or *negative* (“What did you do?“) tone	Brief, gentle pat by PR2’s arm/hand on upper back	To test how verbal tone and touch jointly affect observers’ judgments of the robot’s competence and social qualities	Touch increased morality/fairness ratings (and positive dialogue also raised them) Touch made negative speech seem fairer Perceived skill: higher when the robot touched a male actor; with a female actor, women rated it higher with no touch	Touch improved overall impressions of the robot – it seemed more capable, fair, and caring. Male observers responded more positively, while female observers were less approving, possibly influenced by the robot’s male voice	USA; MTurk online video study. N = 332 (135F/197M; mean age 44; ethnicity NR). People rated PR2 after watching videos where it did vs. did not pat an actor. 400 recruited; 68 excluded (dropout/attention check). Two actors (M/F), demographics NR.
Block et al. (2023)	HuggieBot 2.0/3.0 (autonomous, has touch sensors)	Scripted line that played automatically when near: “Can I have a hug, please?”	Bidirectional touch using an inflatable torso with pressure and microphone sensors and arm torque sensing to detect, classify, and respond to user actions (hold, rub, pat, squeeze)	To clearly signal when the user could start the hug	People liked quick responses, gentle squeezes, small variations in touch, and soft feel; they disliked slow arms, lack of response, awkward hand placement, and having to press a button to initiate the hug	People liked when the robot did not just copy their touch but responded in slightly different ways (felt more natural). The spoken cue helped frame consent by putting the start choice on the user	Germany; lab hugging studies. Total N = 48: Study 1 n = 32 (20F/12M; 21–60, mean 30 ± 7; from 13 countries; ethnicity NR) + Study 2 n = 16 (8F/8M; 22–38, mean 30 ± 4.76; from 10 countries; ethnicity NR). English-speaking community volunteers recruited locally (email/social media/flyers). Builds on the authors’ prior hugging-robot design guidelines and evaluations
Gujran and Jung (2023)	Pepper (pre-programmed – operator triggered, no touch sensors)	Scripted verbal prompts inviting touch (“Shake my hand,” etc.)	Pre-programmed gestures (handshake, fist bump, hug, high five)	To make the robot’s social intentions clear and encourage participants to reciprocate touch	Adding speech greatly increased touch frequency, but no change in perceived likability, animacy, or safety	Verbal prompts clarified intentions and increased engagement in social touch, but participants’ overall perceptions of the robot did not change, potentially influenced by participants’ views of Pepper as “just a machine or computer”	Netherlands; university lab experiment. N = 50 students (20M/27F/3NB; mean age 21.0, SD 2.75; ethnicity NR). 5–10 min chat with Pepper prompting five touch actions; movement-only (n = 25) vs. movement + speech (n = 25). Video used to count how many touches participants reciprocated; Godspeed questionnaire ratings completed afterwards
Jeong et al. (2015)	Huggable – cuddly toy bear (teleoperated, has touch sensors)	Speech was live via Wizard-of-Oz teleoperation: a human operator watched/listened, spoke in real time through the robot with a pitch-shifted voice	Soft, furry body with 12 touch sensors and paw pressure sensors to detect hugs and squeezes. The signals were sent to the operator so the robot could sense when it was being touched and respond appropriately	To provide socio-emotional support for children in a paediatric care context	All children engaged positively through speech and touch; ill children showed more frequent, caring, and emotional interactions, treating the robot like a peer	Touch was key to children’s emotional engagement, especially among sick participants. Future versions should include smarter haptic sensing to recognise different touch types and better support socio-emotional needs. Later hospital-trial results favoured Huggable over tablet avatar/plush conditions (more positive affect/engagement; less sadness; lower reported pain) (Logan et al., 2019)	USA; paediatric hospital research programme evaluating the Huggable teddy-bear robot, underpinned by earlier platform/design papers on touch-sensing “skin” and teleoperation. Evidence centres on Jeong et al. (2015) design + pilot (N = 4; 2 healthy/2 ill) and a later in-hospital comparative/randomised study (robot vs. tablet avatar vs. noninteractive plush; engagement/socio-emotional outcomes) reported in later outputs including Logan et al. (2019), with other papers mainly describing protocol/design or additional analyses
Li et al. (2024)	EmoPus – octopus-shaped soft robot (autonomous, no touch sensors)	LLM-based empathetic dialogue with voice emotion recognition	Tentacle stroking/grasping based on user’s emotional state	To create emotional bonding and mental relief through synchronized verbal and tactile empathy during desk work	Prototype only; no user study	Proposed to reduce stress and loneliness through touch–dialogue empathy	China; university-based prototype development; no user-study setting reported
Nieda et al. (2024)	UR3e robotic arm (pre-programmed – operator triggered, no touch sensors)	Scripted care-giving phrases (“Hello… How are you feeling? Did you rest well?“)	Gentle stroke on right forearm using UR3e arm with 3D-printed hand	To test whether combining stroking with caregiving speech reduces electrical pain more effectively than stroking alone	Stroking with speech was significantly more effective in reducing pain perception than stroking alone	Stroking reduces pain through ‘tactile gating’, while speech boosts positive emotions, which also relieve pain – so combining them leads to stronger overall pain reduction	Japan; university lab experiment. N = 37 students (22M/15F; mean age 23.6; ethnicity NR). Within-subject: electrical forearm pain under nothing vs. robot stroking vs. stroking + caregiving speech; outcome was pain tolerance (max accepted stimulus level)
Sawabe et al. (2022)	UR3 robotic arm (pre-programmed – operator triggered, has touch sensors)	Scripted caregiving phrases (e.g., “Hello, how are you doing?“)	Warm (embedded with heater), gentle stroke on upper back. The arm used a force sensor to keep pressure steady and a temperature sensor to stay close to body warmth	To find out if combining speech with touch makes robot care feel more comforting and human-like	Combining speech and touch raised positive affect and made people see the robot as more human-like	Combining caring speech with gentle touch made the robot’s actions feel more natural and emotionally supportive	Japan; university lab experiment (NAIST). N = 31 Japanese volunteers (12F; other genders NR; mean age 22.8, SD 3.9; ethnicity NR)
Willemse et al. (2017)	NAO v4 (teleoperated, no touch sensors)	Scripted soothing/calming phrases delivered during a scary-movie viewing, at pre-timed interaction moments	Robot’s hand placed on participant’s shoulder during speech during movie	To test whether adding touch impacts stress or social bonding beyond speech alone	No significant benefits from touch on stress, emotion, or perception	Touch was too mechanical and limited; effects may require more natural, and better-timed human-like touch	Netherlands; single-session lab study in a “cozy home-like” room where participants watched scary movies with a NAO robot. N = 39 community adults recruited from a local research participant pool (Touch n = 20; Control n = 19). Age mean 35.72 (SD 9.12; range 19–52). Gender 21F/18M. Ethnicity NR.
Willemse and van Erp (2019)	NAO v4 (teleoperated, no touch sensors)	Scripted calming phrases delivered at pre-timed moments during scary-movie viewing. Beforehand, participants either completed a bonding dialogue with the robot (scripted, responsive-looking dialogue plus gestures/name use) or received only basic familiarisation with its appearance and movements	Robot’s hand placed on participant’s shoulder during speech during movie	To test whether touch adds anything beyond speech (using a similar scary-movie setup to Willemse et al., 2017) and whether robot-initiated touch can elicit positive responses without extensive prior bonding (Unlike 2017, all participants had some prior familiarisation with the robot before the movie.)	Combining touch with speech lowered heart rate and raised intimacy scores	Touch reduced physiological stress and increased perceived intimacy, suggesting touch can strengthen supportive speech. The contrast with Willemse et al. (2017) may reflect prior familiarisation with the robot’s appearance/movements before the movie. However, extensive bonding offered no added benefit over basic familiarisation	Netherlands; single-session lab study where participants watched scary movies with a NAO robot. N = 67 community adults recruited from a research participant database (Touch n = 33; No-touch control n = 34). Age mean 47.9 (SD 20.0; range 18–78). Gender 33F/34M. Ethnicity NR.
Yonezawa et al. (2013)	Wearable stuffed-toy robot (pre-programmed – operator triggered, no touch sensors)	Scripted phrases accompanying either a notification or an affectionate gesture	Pressure, vibration, and warmth applied to the upper arm	To test whether combining speech with tactile cues makes robot communication clearer and more emotionally expressive	Adding touch resulted in participants reporting that messages were easier to understand and that they felt more affection towards the robot	Touch helps communication feel clearer and more caring. But it only works well if the strength and timing of the touch are comfortable	Japan; laboratory study (single-session, standing task with a wearable upper-arm robot delivering brief scripted touch + speech). N = 26 young adult volunteers (likely university sample). Age: 19–25 (mean ± SD NR). Gender: 13F/13M. Ethnicity NR.
Zhang et al. (2025)	Pepper (pre-programmed – operator triggered, no touch sensors)	Scripted phrases (in a human-like or mechanical counselling voice)	Gentle hand-hold during conversation (Pepper’s hand was lowered onto the participant’s hand)	To test how touch and voice style (human-like vs. mechanical) jointly influence self-disclosure and attachment in AI counselling	Touch increased both self-disclosure and attachment. The overall effect on disclosure was stronger with the human-like voice than with the robotic voice	Touch boosted emotional bonding and perceived empathy, especially with the human-like voice	China; university lab simulated counselling study (single session ∼10–15 min). N = 30 university participants (students; all unfamiliar with the robot). Age 20–33 (mean 25.07, SD 2.77). Gender 16M/14F. Ethnicity NR.

One reason for this may be that touch interaction is challenging to design, carrying physical, psychological, and socio-cultural implications (Chen et al., 2014; Buono et al., 2025). It is context-dependent and often highly ambiguous (Price et al., 2022), and integrating touch with other modalities introduces major engineering challenges in sensing, timing, and synchronisation (Emami et al., 2024). These difficulties are compounded by safety concerns and the cost of hardware development and testing, particularly in a market where many promising social robotics initiatives have failed to achieve sustained commercial success, despite encouraging academic proof-of-concept work (Tulli et al., 2019).

This divide is evident in existing systems. Among tactile-focused platforms, Haptic Creature (research prototype) is a small, cushion-sized robotic “pet” with a vibrotactile “purr” motor that activates in response to how the user touches, strokes, or handles it, modulating vibration strength and pattern based on the robot’s so-called “internal emotional state” (Yohanan, 2012; Yohanan and MacLean, 2012). However, it lacks any capacity to interpret or adapt to verbal emotional cues, or to talk with the user. Similarly, while LOVOT (commercial robot, mentioned earlier) excels at tactile interaction, it relies entirely on non-verbal communication (Dinesen et al., 2022; Imamura et al., 2023). A study with animal-like companions for older adults underscores this gap between tactile interaction and users’ desire for verbal engagement. Although the robots were non-speaking, participants often sought verbal interaction, and, as one put it, *“you will not be much use to me if you do not talk to me”* (Bradwell et al., 2019).

Conversely, conversational AI systems like Ellie (research virtual agent) rely on input modalities such as facial expressions, voice tone, and posture in addition to speech content to generate empathic dialogue, but they lack physical embodiment for producing tactile output (Rizzo et al., 2016). A few promising efforts bridge this gap. Huggable (research platform), for example, is a plush robotic bear designed for paediatric care that pairs dialogue with physical responsiveness; it can speak, detect when a child hugs it, and gently reciprocate the embrace through internal actuators beneath its soft body (though teleoperated) (Stiehl et al., 2005; Logan et al., 2019). Such systems illustrate the potential of combining modalities, yet the field remains fragmented and underexplored (Figure 1).

### 1.4 Aim of this scoping review

Therefore, to advance the design of empathic social robots, a clearer understanding is needed of how to combine speech with affective forms of intentional touch–such as a hug, a stroke, or a gentle vibration–that are intended not only for practical purposes, but to shape users’ emotional and relational experience. To our knowledge, this is the first review to examine how speech and touch have been combined in robots used for social HRI.

As this is a scoping review (i.e., mapping what exists in a research area), our objective is to identify and catalogue all known robots that combine spoken language and intentional touch for social or emotional interaction. As such, we pose broad, mapping-oriented research questions rather than a single, narrowly defined research question. Specifically, we aim to address the following research questions.

1. What robots used for social HRI employ touch as an affective or social design resource alongside language?
2. How are speech and touch coordinated within these robots?
3. What effects does combining speech and touch have on participants, compared with speech-only or touch-only HRI?
4. What design factors appear to enable or constrain effective speech–touch HRI, and what gaps remain in current approaches?

By synthesising existing implementations and findings, this review seeks to inform the design of future multimodal social robots (and potential upgrades to existing platforms). Such responsive systems may hold particular promise in care settings, including mental health support, care for older adults, and paediatric contexts, where coordinated speech and touch could enhance emotional support and improve wellbeing.

## 2 Methods

This review follows Preferred Reporting Items for Systematic Reviews and Meta-Analyses extension for Scoping Reviews (PRISMA-ScR) (Tricco et al., 2018) and the six-stage Arksey and O’Malley framework (Arksey and O'Malley, 2005), including defining the research question, identifying relevant studies, selecting studies, charting data, and summarising findings.

### 2.1 Eligibility criteria

#### 2.1.1 Inclusion criteria

We include records of real, physically implemented robots used in social or emotional interaction with humans (i.e., ‘social robots’ in this review), regardless of the application domain. Eligible robots communicate via spoken or text-based language (prerecorded or generated) and also actively produce or invite physical contact (e.g., saying “Can I have a hug?” while opening their arms), for affective HRI. We placed no restrictions on study design, the presence of a documented user study or interaction context (for example, laboratory or home-setting). Robots could be autonomous or teleoperated.

In neuroscience, affective touch is often used in a relatively narrow sense to refer to slow, gentle stroking on hairy skin at speeds considered optimal for activating C-tactile (CT) fibres–a class of sensory nerve fibres in the skin tuned to gentle, caress-like stroking (McGlone et al., 2014). In this review, however, we use the term more broadly and in a theory-neutral way to refer to touch that evokes feelings or emotional responses. Our focus is therefore on the socio-emotional effects of touch, such as comfort, reassurance, affiliation, and care. This broader definition includes CT-targeted stroking as one example, but also encompasses more recognisable social touch gestures, such as stroking, patting, holding, hugging, and hand-holding, as well as simpler tactile comfort cues such as warmth, gentle pressure, and vibration.

#### 2.1.2 Exclusion criteria

We excluded systems that used only one of the two target modalities, that is, language without touch or touch without language. We also excluded robots in which physical contact served solely to accomplish a functional task and was not designed, framed, or evaluated as part of socio-emotional interaction. This included contact used only to guide, support, mobilise, or hand over objects, rather than to convey comfort, reassurance, affiliation, or care. We recognise that rehabilitation and other care contexts may still involve important social and emotional dimensions; our exclusion criterion therefore concerned not the broader setting, but whether touch was treated as an affective or relational component of the interaction.

We also exclude sources that are conceptual only, not in English, or lacking sufficient detail on how speech and touch are combined during interaction. Robots that produce only non-linguistic vocalisations without language, for example, LOVOT and PARO (Petersen et al., 2017; Dinesen et al., 2022), were also excluded, as our focus was specifically on systems that paired linguistic verbal output with touch intended to shape the user’s socio-emotional or relational experience.

### 2.2 Search strategy and sources

The search strategy was structured around four key concepts: *Robot*, *Verbal Interaction*, *Tactile Interaction*, and *Affective/Social Interaction*. Within each concept, related terms were combined with OR, and the four concepts were linked with AND. Searches were conducted on 30 July 2025 across five databases: IEEE Xplore, PubMed (MEDLINE), ACM Digital Library, Web of Science, and Scopus. Search strings were adapted to each database’s syntax, and full queries with result counts are available in Supplementary Appendix SA. Because many robots are not indexed, searches were also conducted via Google, focusing on known systems, company websites, and demos to capture additional implementations. These sources supplemented, but did not replace, peer-reviewed evidence.

### 2.3 Selection of sources of evidence

Records were deduplicated in EndNote, then screened in R. One reviewer manually screened all titles and abstracts, with GPT-4.1 (via the GPTscreenR package) (Wilkins, 2023) independently screening approximately 50% of records to assess consistency, and the reviewer verified discrepancies. This dual-screening approach aligns with emerging evidence supporting large language model (LLM)-assisted screening (Sanghera et al., 2025). Full texts were assessed by the same reviewer. During title-and-abstract screening, we excluded records that were additional reports of robot implementations already represented, to avoid double counting at the system level, since we report the number of distinct robot implementations that combine speech and touch (these reports were still consulted and cited where appropriate in the review).

### 2.4 Data charting

Table 1 summarises the information charted from each included implementation, including the robot, dialogue component, touch component, purpose of combining modalities, key results, authors’ interpretation, and evidence context. Data were initially extracted and recorded by one reviewer using a structured charting table. To strengthen rigour, all entries in Table 1 and the corresponding results summaries were subsequently checked by a second reviewer against the original sources for accuracy. When the same design was evaluated across multiple studies, those studies were grouped under a single robot entry.

### 2.5 Synthesis method

We narratively synthesised the findings across studies, grouping them into thematic categories based on the type of multimodal interaction examined. Specifically, we distinguished between studies testing speech-only versus speech + touch interactions, those testing touch-only versus touch + speech, and non-comparative studies that integrated both modalities without direct contrast conditions. Within each theme, we examined participants’ behavioural, physiological, and emotional responses to the robot, along with contextual factors such as how the robot communicated and how its touch felt to users. For comparative studies, we conducted a descriptive assessment of methodological rigour, focusing on the clarity of the speech–touch contrasts and the strength of the evidential support for reported outcomes.

## 3 Results

### 3.1 Study selection

**FIGURE 2:**
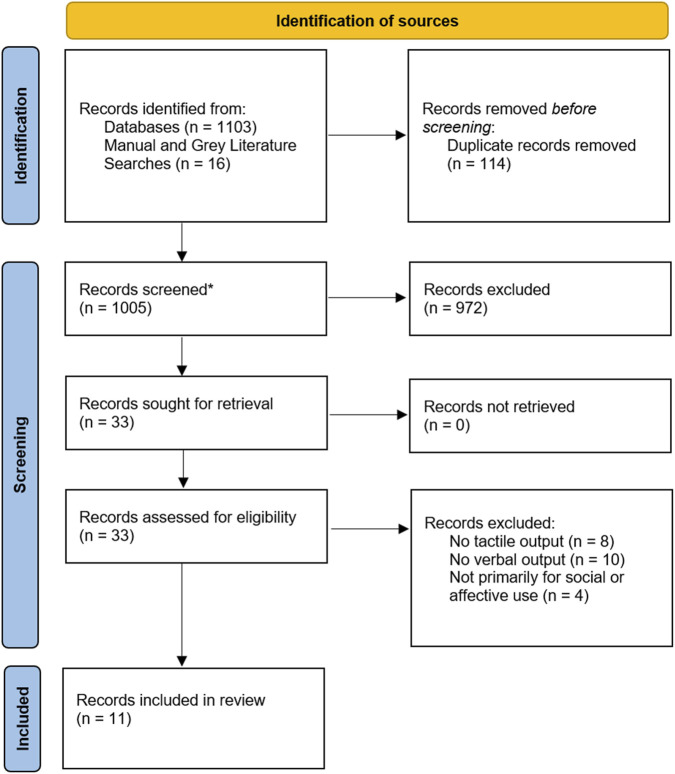
PRISMA Scoping review flow diagram of record selection for distinct robot implementations that combine speech and touch.

Database searches retrieved 1,103 records, with 16 additional items identified via web searches. After deduplication, 1,005 unique records remained. Following title and abstract screening, thirty-three full texts were assessed for eligibility. Following full-text screening 11 sources met all criteria for inclusion, each mapping to a functionally distinct HRI system (Figure 2).

### 3.2 Characteristics of included studies

#### 3.2.1 Study designs and modality contrasts

Eleven studies described eleven robot implementations that integrated linguistic output with active or invited touch. Most studies were comparative laboratory experiments using convenience samples, examining how the addition of touch or speech influenced interaction outcomes. Specifically, five studies contrasted speech-only with speech-plus-touch (Yonezawa et al., 2013; Willemse et al., 2017; Arnold and Scheutz, 2018; Willemse and van Erp, 2019; Zhang et al., 2025); three contrasted touch-only with touch-plus-speech (Sawabe et al., 2022; Gujran and Jung, 2023; Nieda et al., 2024). The remaining three included no controlled modality contrast in the selected paper (or in other publications on the same robot that we located). These comprised a paediatric deployment using Wizard-of-Oz control (i.e., a hidden human operator controlled the robot’s responses) (Jeong et al., 2015), an autonomous hugging evaluation where speech served only as a ‘consent’ cue (Block et al., 2023), and a design prototype without user testing (Li et al., 2024).

#### 3.2.2 Robot types and embodiments

**FIGURE 3:**
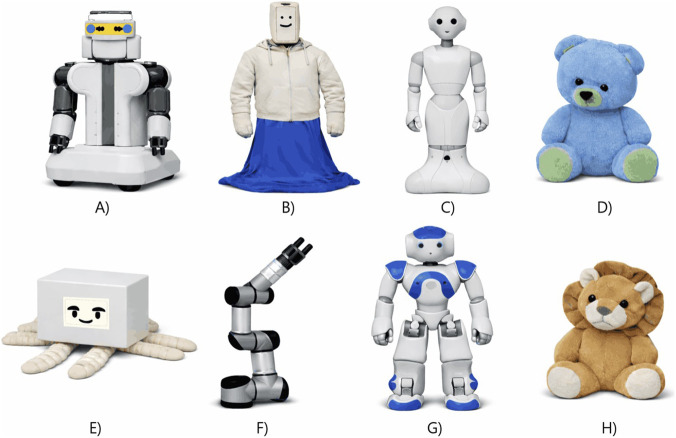
Illustrative representation of the robot implementations included in this review (not exact, like-for-like reproductions). **(A)** PR2; **(B)** HuggieBot; **(C)** Pepper; **(D)** Huggable; **(E)** EmoPus; **(F)** UR3/UR3e; **(G)** NAO; **(H)** wearable tactile companion robot. Images were produced with the aid of GPT-Image 1.5 to provide a general visual impression of what the robots looked like.

Across the eleven implementations, robot types included repurposed humanoid/service robots (PR2, NAO, Pepper; n = 5), industrial robotic arms (UR3/UR3e; n = 2), custom soft/social robots (Huggable, HuggieBot, EmoPus; n = 3), and a custom wearable tactile companion robot (n = 1). Overall, most implementations used repurposed rigid platforms (7/11), with fewer purpose-built soft or wearable systems. Figure 3 presents an illustrative representation of the included robot implementations.

#### 3.2.3 Dialogue implementation

Across the evidence base, the dialogue component was typically limited to short, pre-scripted utterances (delivered autonomously or via teleoperation); only one system reported LLM-based, AI-generated dialogue (Li et al., 2024).

#### 3.2.4 Touch types

Only two studies explicitly targeted ‘affective touch’ in the narrow neuroscience sense (slow, gentle stroking designed to engage the skin’s CT fibres–special touch fibres thought to support the pleasant, comforting feeling of a gentle caress (Sawabe et al., 2022; Nieda et al., 2024). All remaining implementations used touch or tactile cues in a broader social/affiliative sense (e.g., hand-holding, hugging, patting, warmth, pressure, or vibration).

#### 3.2.5 Why speech and touch were combined

Across the eleven systems, speech and touch were combined for three main purposes: to provide affective support, including comfort, bonding, and pain or stress reduction (Jeong et al., 2015; Sawabe et al., 2022; Li et al., 2024; Nieda et al., 2024; Zhang et al., 2025), to clarify social affordances and consent for touch by signalling when and how touch was appropriate (Yonezawa et al., 2013; Block et al., 2023; Gujran and Jung, 2023), and to shape social evaluations and moral impressions of the robot, such as perceived warmth, care, and fairness (Willemse et al., 2017; Arnold and Scheutz, 2018; Willemse and van Erp, 2019).

#### 3.2.6 Geographic distribution of studies

Geographically, the studies were distributed across the Netherlands (n = 3) (Willemse et al., 2017; Willemse and van Erp, 2019; Gujran and Jung, 2023), Japan (n = 3) (Yonezawa et al., 2013; Sawabe et al., 2022; Nieda et al., 2024), China (n = 2) (Li et al., 2024; Zhang et al., 2025), the United States (n = 2) (Jeong et al., 2015; Arnold and Scheutz, 2018), and Germany (n = 1) (Block et al., 2023). Several studies recruited university participants, online panel participants, or community volunteers (Yonezawa et al., 2013; Willemse et al., 2017; Arnold and Scheutz, 2018; Willemse and van Erp, 2019; Sawabe et al., 2022; Block et al., 2023; Gujran and Jung, 2023; Nieda et al., 2024; Zhang et al., 2025). Overall, the evidence is not Western-only, but limited demographic reporting and a small, heterogeneous evidence base prevent meaningful assessment of representativeness, such as whether the literature is skewed toward Western, Educated, Industrialised, Rich, and Democratic (WEIRD) populations (Henrich et al., 2010), or analysis of cultural and demographic moderators.

Key characteristics and findings for each of the eleven robot implementations appear in Table 1.

### 3.3 Evaluation methodologies

The strategies used to evaluate these systems were heterogeneous. Most studies relied on self-report questionnaires, utilising either validated scales for impressions of the robot and user emotion (Willemse et al., 2017; Logan et al., 2019; Willemse and van Erp, 2019; Gujran and Jung, 2023; Zhang et al., 2025) or custom items assessing constructs like comfort, trust, and competence (Yonezawa et al., 2013; Arnold and Scheutz, 2018; Block et al., 2023). Notably, unlike the other comparative studies, Arnold and Scheutz (2018) relied on observer ratings from video rather than ratings from the people actually interacting with the robot. Others relied on single-item ratings of human-likeness (Sawabe et al., 2022) or pain tolerance thresholds (Nieda et al., 2024). Beyond self-report, researchers analysed objective behaviours, including how often participants touched the robot (Block et al., 2023; Gujran and Jung, 2023), speech sentiment (Logan et al., 2019), and willingness to donate money or time (Willemse et al., 2017; Willemse and van Erp, 2019). Finally, a smaller number of studies integrated physiological measures, ranging from skin conductance and heart rate (Willemse et al., 2017; Logan et al., 2019; Willemse and van Erp, 2019) to facial muscle activity (Sawabe et al., 2022) and brain activity (Zhang et al., 2025).

### 3.4 Methodological rigour of comparative studies

Across the five studies comparing speech-only with speech-plus-touch, rigour was moderate overall. The speech–touch contrast was well controlled, with scripted speech held constant and touch added in predefined ways, allowing reasonably clean causal comparisons; however, one study used a video vignette in which participants judged touch they observed, rather than touch they experienced (Arnold and Scheutz, 2018). Evidence is limited because touch was typically brief and highly scripted and interactions were single-session laboratory tasks.

Across the three studies comparing touch-only with touch-plus-speech, rigour was also moderate. Each used a controlled laboratory contrast in which touch behaviour was held constant and speech was added, supporting causal interpretation, though evidence was based on short, single-session tasks and highly scripted interactions.

### 3.5 The impact of adding touch to speech

#### 3.5.1 Core pattern across studies

The most consistent finding across studies was that robot-initiated touch during speech can lead to more positive social appraisals of the robot. Here, “robot touch” refers to brief, robot-initiated physical contact delivered alongside short, scripted, socially supportive speech (e.g., a hand placed on or lightly tapping the shoulder, back, or hand, or a wearable providing warmth/pressure/vibration to the upper arm). In these speech-plus-touch studies, touch was not implemented as CT-targeted stroking, but as broader social/affiliative contact. See Table 1 for a summary of touch types and the accompanying speech used across studies.

Specifically, four of five studies adding touch to speech (Yonezawa et al., 2013; Arnold and Scheutz, 2018; Willemse and van Erp, 2019; Zhang et al., 2025) reported improved evaluative psychosocial outcomes, including more favourable impressions of the robot (e.g., caring, fairness/morality), stronger relational outcomes (e.g., intimacy/attachment), and–in counselling contexts–greater willingness to self-disclose. In contrast, Willemse et al. (2017) reported no significant benefits of adding a brief shoulder touch to soothing speech, which the authors attributed to the cold, rigid feel of the contact.

#### 3.5.2 PR2 back-pat during workplace task

Arnold and Scheutz (2018) found that, in a staged workplace-style computer task where something briefly goes wrong and a robot responds, adding a brief, gentle robot-initiated pat on the upper back (delivered while speaking) significantly elevated participants’ Likert-scale ratings of the PR2 (Figure 3A) as caring, fair, and guided by good values. They also found that encouraging speech produced higher ratings than a critical line. When the robot used a critical line, adding the pat increased fairness ratings compared with the same words delivered without touch. The authors suggest the pat helped because touch can carry an effect independent of speech (often interpreted as a pro-social cue in context) and may, in some cases, mitigate or soften the impact of negative tone. Unlike the other comparative studies in this review, however, this was an online video-perception study: participants watched the interaction and rated it, so the effects reflect observers’ perceptions, not the experiences of people who were directly touched.

#### 3.5.3 Pepper hand-holding in a counselling context

Similarly, in a counselling scenario, Zhang et al. (2025) found that robot-initiated hand-holding–Pepper (Figure 3C) reaching out to hold the participant’s hand–increased willingness to self-disclose and feelings of attachment to Pepper robot. This was during a conversation about work and study stress (delivered in a lab). Attachment rose partly via higher perceived empathy (an effect the authors noted explained about two-fifths of the overall increase). Voice-type mattered for disclosure: touch significantly increased self-disclosure only when paired with a human-like (anthropomorphic) voice, not a robotic-sounding one. For attachment, touch improved participants’ attachment ratings under both voices. The touch-related increase in attachment (relative to no-touch) was larger with the mechanical voice, but overall attachment remained highest when the human-like voice was combined with physical touch. Zhang et al. (2025) suggest this is because a human-like voice raises expectations–and since Pepper’s hand felt cold and rigid, the touch seemed less genuine. They note the attachment effects of touch might improve if the robot’s hands were softened or warmed (e.g., fabric cover or heated pads).

#### 3.5.4 NAO shoulder touch during frightening movie

Beyond subjective appraisals, adding touch to speech demonstrated measurable physiological and behavioural effects (Willemse and van Erp, 2019). NAO (Figure 3G) delivered calming phrases while participants watched frightening films. When this speech was paired with gentle shoulder touches, participants’ heart rate decreased, whereas heart rate was higher in the speech-only condition, suggesting a calming effect of touch. The authors also found higher intimacy ratings in the speech + touch condition. The authors interpret this as touch acting as a supportive nonverbal cue that can reduce stress. In an earlier paper, Willemse et al. (2017) did not observe these touch-related benefits in a similar scary-movie experimental setup, and suggested that contextual factors–such as brief familiarisation or rapport with the robot beforehand–might help explain the discrepancy.

#### 3.5.5 Wearable haptics during outings

**FIGURE 4:**
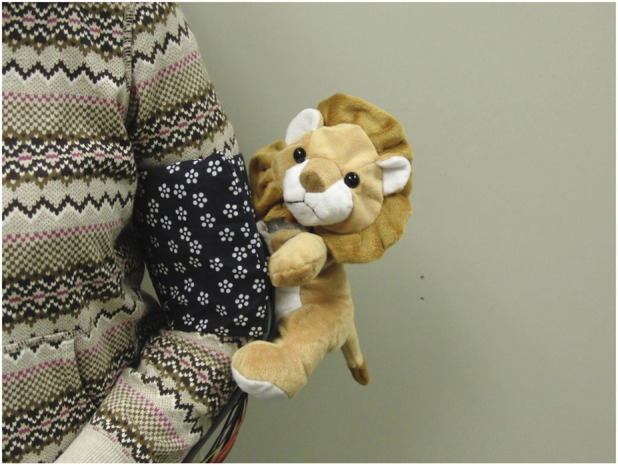
Wearable stuffed-toy robot attached to the upper arm (photograph © Tomoko Yonezawa and Hirotake Yamazoe, used with permission).

Yonezawa et al. (2013) built a wearable upper-arm companion robot (Figure 4) that used touch in two ways: a brief tap + vibration to get attention before speaking, and a gentle inflatable squeeze + warmth to convey comfort/affection. It looked like a small plush lion (Figure 3H) attached to the outside of a blood-pressure–style arm cuff (so it appears to “hug” the arm); the pressure/vibration/temperature hardware sat in the cuff against the skin, while the plush provided the “face/body” of the robot. The “tap” involved the plush paw/arm visibly moving, but the felt tapping sensation was delivered by the cuff’s vibration motor timed to that motion.

The intended use was to support older adults during outings (e.g., walks or errands), and the system was evaluated in a lab simulation. Adding these tactile cues alongside speech made messages easier to notice and understand and increased users’ self-reported affection toward the robot. Yonezawa et al. (2013) suggest the benefit comes from treating touch as a caregiver-like nonverbal cue: physical contact can directly convey closeness and affection, and it can also support spoken communication by drawing attention and making the interaction feel more natural.

#### 3.5.6 Moderators and null findings

However, the benefits of adding touch are not universal. Arnold and Scheutz (2018) identified important moderating factors, notably gender, where effects on perceptions of the robot’s capability depended on the gender of the person being touched and the gender of the observer. Women rated it lower when being touched; however, the authors noted that the robot had a male voice, which may have influenced these perceptions.

Willemse et al. (2017) used a similar setup later revisited by Willemse and van Erp (2019) (see Section 3.5.4), where NAO delivers calming phrases during frightening films, sometimes adding a shoulder touch. But in Willemse et al. (2017), adding the touch did not help–there were no clear changes in physiology, self-reported affect, stress, or prosocial behaviour compared with speech alone. The authors suggested this may be because of the “mechanical appearance and feel of the touch” – a brief pat on the shoulder with a “cold” plastic hand at room temperature (20 °C) rather than a warm, gentle stroking motion. They suggested warming the hand to human skin temperature (around 32 °C) could improve outcomes, and noted that the cold, rigid feel potentially evoked an “Uncanny Valley” response, since the plastic hand resembled a human hand visually but lacked the soft warmth of human touch, creating an unsettling impression. When the study was revisited in 2019 (Willemse and van Erp, 2019), the touch implementation was not described as warmer or more human-like, but all participants had prior exposure to the robot’s appearance and movements before the movie. Willemse and van Erp (2019) suggest this familiarisation may have helped relative to introducing robot-initiated touch without prior exposure.

### 3.6 The impact of adding speech to touch

#### 3.6.1 Core pattern across studies

Conversely, several studies examined the impact of augmenting a tactile interaction with spoken language. Two of three studies adding speech to touch (Sawabe et al., 2022; Nieda et al., 2024) reported improved evaluative psychosocial outcomes (Sawabe: higher positive valence and human-likeness; Nieda: greater pain tolerance). Across these “speech-added-to-touch” implementations, speech consisted of short, pre-scripted phrases tightly coupled to the touch event (rather than sustained dialogue), and touch comprised simple, repeatable gestures, including gentle stroking of the back or forearm (CT-targeted in Sawabe et al., 2022; Nieda et al., 2024) and discrete social gestures (e.g., handshake, high five, hug in Gujran and Jung, 2023; see Table 1 for details).

#### 3.6.2 Caregiving speech paired with UR3/UR3e stroking

Sawabe et al. (2022) demonstrated that pairing gentle stroking of participants’ upper/mid back (over clothing) using a UR3 (Figure 3F) robotic arm with a warmed touch surface, alongside simple caregiving phrases, increased self-reported positive valence and perceived human-likeness compared with touch alone. The stroking was carefully standardised to what is described as CT-optimal parameters (i.e., speeds most likely to activate C-tactile fibres, which are associated with pleasant affect), approximately 5 cm/s over 15 cm for 10 s at around 3 N. The content of speech was based on samples of caregivers speaking in real-life care environments, although the experiment itself was lab-based, and the authors did not report detailed characteristics of the synthesised voice beyond using text-to-speech. Physiologically, touch-plus-speech produced higher zygomaticus major activity (the “smile” muscle), indicating a more positive affective response. They suggest speech frames the touch as caring and more human-like, which may amplify the positive emotional effect of the CT-style stroking they performed.

Nieda et al. (2024) similarly found that when a robot (UR3e; Figure 3F) paired forearm stroking with caregiving speech (a prerecorded female voice intended to mimic a nursing/care scene), participants tolerated higher levels of experimentally induced electrical pain than during stroking alone. The stroking was carefully controlled (right forearm; ∼1 N; 100 mm/s = 10 cm/s; 100 mm stroke path) and the authors explicitly describe this speed as optimal for activating CT fibres; they do not report the touch being warmed/heated (i.e., no temperature manipulation is described). They interpret the added benefit of speech as coming from two complementary routes: tactile “gating” (gentle touch inhibiting pain signalling) plus speech-evoked positive affect, which can further reduce how intense the pain feels.

#### 3.6.3 Pepper verbal invitations to touch

Speech also helps people know when and how to engage in touch. In Gujran and Jung (2023), the robot’s (Pepper; Figure 3C) movement-only cues (hand extended, arms open) failed to elicit touch. Adding clear verbal invites – “Let me shake your hand,” “That deserves a high five,” “Let me give you a hug” – greatly increased reciprocation. Speech can therefore provide the social affordances and permission that make robot-initiated touch understandable and actionable. However, this study found that touch did not influence ratings of the robot’s likability, intelligence, or anthropomorphism. The authors suggested this was because the positive emotional impact of robot touch may be relatively weak in short encounters and easily overshadowed by pre-existing views of robots as machines.

### 3.7 Non-comparative studies

#### 3.7.1 Overview and shift to custom social robots

Three studies examined robots that integrate speech and touch without directly comparing these modalities. Whilst the robots discussed above are primarily repurposed laboratory robots (i.e., UR3, UR3e, and Pepper), the robots discussed below are more custom designed for affective and social interaction, emphasising soft materials, and comforting touch, rather than general-purpose functionality.

#### 3.7.2 HuggieBot hugging interaction

Block et al. (2023) evaluated HuggieBot (Figure 3B), a full-body warmed hugging robot that initiates interaction through a verbal prompt (“Can I have a hug?”); after which the robot senses and classifies user gestures (hold, rub, pat, squeeze) and responds accordingly. In tactile terms, it delivers warmth, a soft/compliant body, and adaptive hugging pressure. The robot uses a real-time gesture classifier based on its torso signals, then applies a probabilistic behaviour policy that chooses responses based on which reactions people previously liked most, with some added variety so it does not feel mechanical. Participants reported that strict mirroring–where the robot always copies the user’s exact gesture (e.g., rub→rub, pat→pat, squeeze→squeeze) – felt mechanical, whereas a little spontaneity and variety made the interaction feel more natural and socially responsive. After repeated hugs, users found the robot more natural, more enjoyable, and more socially intelligent, and they felt more understood.

The design of HuggieBot was informed by earlier hugging-robot design guidelines, which emphasise the importance of soft, warm (approximately human-like temperature) surfaces alongside responsive and adaptive hugging behaviours (Block et al., 2021). These guidelines draw in part on findings reported by Block and Kuchenbecker (2019), which showed that when participants experience multiple types of robot hugs, adding softness and warmth can increase perceived safety and comfort.

#### 3.7.3 EmoPus LLM-based dialogue and tactile interaction

Li et al. (2024) introduced EmoPus (Figure 3E), a soft, octopus-shaped desk companion that integrates LLM-based voice dialogue with cable-driven tentacles capable of providing tactile comfort (e.g., curling or resting on the user’s hand). The system incorporated basic affect sensing, using “speech emotion recognition” – likely prosody-based (i.e., how it’s said, e.g., pitch) rather than lexical (i.e., what words were said), though details were not reported–to infer emotional tone from the user’s voice and adapt its dialogue and tentacle behaviour accordingly. A Grove Vision AI module (a vision sensor) and 24 GHz mmWave sensor (a small radar sensor) were used to detect user presence and simple “visual cues related to emotion”. However, the authors did not specify which emotions the system recognised or report any details of model features. The paper documented a working prototype but reported no user study or evaluation.

#### 3.7.4 Huggable plush hospital robot

Finally, Jeong et al. (2015) describe Huggable (Figure 3D), a plush teddy-bear robot designed to comfort children in hospital. Huggable is covered in a full-body “sensitive skin” with twelve capacitive touch sensors (earlier versions had over 1,500 embedded sensors (Stiehl et al., 2008)) enabling it to feel touches and gestures across its surface, while its removable fur helps preserve the robot’s “warm and fuzzy appeal”. The robot is teleoperated by a human controlling its movements and speech–which was pitch-shifted (a Wizard-of-Oz setup). The teleoperator saw and heard the child through an Android smartphone embedded in Huggable (using its camera and microphone).

Unlike the other systems that were predominantly evaluated only in laboratory settings, Huggable was also tested in a randomised controlled trial conducted in a hospital that compared it with a tablet-based avatar and a noninteractive plush bear amongst children (aged 3–10) (Logan et al., 2019). Children interacting with Huggable displayed greater positive affect, more joyful speech, less sadness, and longer engagement, and parents’ proxy ratings suggested that they believed their children were in less pain after the Huggable session.

## 4 Discussion

The following sections provide a critical synthesis of the reviewed literature on the integration of speech and touch in HRI systems and the potential implications of these findings for interaction design.

### 4.1 Conditions under which speech–touch integration appears beneficial

Taken together, these studies indicate that incorporating speech and touch in human–robot interaction can enhance affective and psychosocial outcomes relative to unimodal baselines in some contexts. More modalities were not necessarily better; benefits depended on how naturally and appropriately speech and touch were combined.

In this evidence base, the studies that reported positive psychosocial/affective effects typically involved scenarios where.

1. The interaction context calls for socio-emotional support (e.g., stress, fear, counselling, caregiving, easing discomfort for paediatric patients) (Yonezawa et al., 2013; Jeong et al., 2015; Arnold and Scheutz, 2018; Willemse and van Erp, 2019; Sawabe et al., 2022; Nieda et al., 2024; Zhang et al., 2025).
2. Speech provides an interpretable social frame for the tactile act (e.g., reassurance, caregiving intent that makes the touch clearly interpretable as support) (Yonezawa et al., 2013; Arnold and Scheutz, 2018; Willemse and van Erp, 2019; Sawabe et al., 2022; Nieda et al., 2024; Zhang et al., 2025).
3. The touch itself is physically pleasant and expectation-consistent (e.g., warm, soft, non-threatening; avoiding cold/rigid/uncanny-feeling contact, and managing expectations via familiarisation when needed) (Yonezawa et al., 2013; cf. Willemse et al., 2017; Willemse and van Erp, 2019; Sawabe et al., 2022; Block et al., 2023; Zhang et al., 2025).

Benefits of combining speech and touch were observed both when adding speech to an existing tactile interaction–particularly when the tactile component involved CT-targeted gentle stroking on the forearm or back (Sawabe et al., 2022; Nieda et al., 2024) – and when adding touch to an existing spoken interaction (Yonezawa et al., 2013; Arnold and Scheutz, 2018; Willemse and van Erp, 2019; Zhang et al., 2025).

### 4.2 Touch quality, embodiment, and affective experience

Notably, the two studies in which adding speech to touch produced clear benefits both used “CT-targeted stroking” (Sawabe et al., 2022; Nieda et al., 2024). This refers to gentle, caress-like stroking designed to preferentially activate C-tactile sensory fibres in the skin. CT neurons are a specialised subtype whose sensory fibres do not respond strongly to stimuli such as pressure, but are tuned to light, gentle stroking and are associated with soothing, pleasant feelings (McGlone et al., 2014). Such gentle stroking is believed to support bonding and calming. CT responses are also strongest for skin-like warmth, aligning with temperatures typical of human touch (Ackerley et al., 2014).

While CT-targeted stroking is one plausible route to affective benefits, many HRI touch behaviours (e.g., brief pats or vibrotactile feedback) are not CT-specific, so effects will reflect broader mechanisms and depend on how contact is implemented. When we focus on tactile feedback properties rather than recognisable social touch gestures, abstract cues such as vibration were not used in isolation in the included studies; instead, they were either combined with other tactile cues (e.g., warmth and pressure) or embedded within broader touch acts (e.g., Yonezawa et al., 2013; Sawabe et al., 2022; Block et al., 2023). This may be because abstract tactile feedback can be subtle and highly ambiguous for participants (Price et al., 2022), and therefore may lack clear affective or social meaning. Accordingly, if designers rely primarily on haptic feedback rather than recognisable social touch, combining vibration, pressure, and temperature (as in Yonezawa et al., 2013), may help reduce the risk that the interaction is experienced as undifferentiated sensory stimulation (such as vibration alone) rather than meaningful touch. Vibration, pressure, and temperature may be especially important features to prioritise in emotion-discriminative haptic design, given their links to skin receptors and the evidence for strong links between thermal skin changes and emotion (Price et al., 2022).

More generally, touch that feels natural and warm may better support comfort, trust, and perceived empathy, whereas brief, cold, ambiguous, or mechanically imposed contact may offer little or no added value (Willemse et al., 2017; Gujran and Jung, 2023). For example, the study that informed the design of HuggieBot found that when participants experienced multiple types of robot hugs, adding softness and warmth increased perceived safety and comfort (Block and Kuchenbecker, 2019).

These findings can be interpreted through a soma design lens (from *soma*, the Greek word for *body*) which puts bodily sensation at the centre of affective experience (Höök, 2018). Soma design, in essence, attends to how an interaction feels in the body–because whether touch is supportive is often hard to specify and may simply come down to whether “it just feels right”. Rather than treating interaction as purely cognitive, this perspective emphasises felt, bodily experience (e.g., warmth and softness) as central to affective response. It contrasts with rapid, “rational” design processes that often dominate technology development, instead encouraging designers to reflect carefully on how an interaction feels, including subtle details (such as material type) that may seem negligible on paper but can meaningfully influence bodily and emotional responses. Alongside tactile properties, contextual factors also matter, as even minimal familiarisation with a robot’s presence and movements may shape whether robot-initiated touch feels calming (Willemse and van Erp, 2019). Similarly, after repeated hugs with HuggieBot, users rated the robot as more natural, enjoyable, and socially intelligent, and reported feeling more understood (Block et al., 2023).

### 4.3 Implications for healthcare and assistive care robots

Across the included studies, healthcare-relevant use cases involved counselling-style support (Zhang et al., 2025), caregiving reassurance (Sawabe et al., 2022; Nieda et al., 2024), stress reduction (Willemse et al., 2017), and comfort in hospital settings (e.g., paediatric support) (Jeong et al., 2015; Logan et al., 2019). Beyond contexts where the primary aim is socio-emotional support, there may also be value in applying socio-emotional speech–touch principles to robots whose main role is physical assistance rather than primarily social interaction. This includes systems assisting with lifting or moving people (e.g., Jiang et al., 2015) or rehabilitation, where the primary goal is functional but the way contact (and accompanying dialogue) is delivered may still influence comfort, dignity, trust, and acceptance. We found little evidence of physical rehabilitation or manipulation robots in which touch was explicitly treated and evaluated, alongside speech, as an affective interactional resource within the user’s socio-emotional or relational experience. This suggests a research gap in extending socio-emotional design to speaking care robots that can, for instance, recognise how a patient is feeling and adjust their words accordingly in a supportive, caring manner (Tamantini et al., 2023), while also treating contact itself as an affective interactional resource, for example, through its thermal, material, and force characteristics (where feasible).

### 4.4 Future directions for embodied speech–touch AI

Given the review’s focus on the impact of language when augmented with touch via robots, it is notable that only one system (Li et al., 2024) employed an LLM-based approach to language generation. This likely reflects both the timing and aims of the literature, as many implementations predate current-generation LLMs. Where generative dialogue would be desirable, engineering safe, autonomous speech–touch behaviour introduces substantial complexity for the largely laboratory-based, proof-of-concept systems represented in this review. Nonetheless, future social agents are likely to adopt more flexible and adaptive language generation, particularly given the potential for LLM-based systems to support interactions experienced as empathic through more personalised dialogue (Howcroft and Blake, 2025).

Embodiment and touch may represent an important direction for advancing empathic AI in healthcare and related settings where physical presence and social touch are appropriate. This does not diminish the value of virtual chatbots, which remain a scalable means of providing support across a broad spectrum of uses (Laymouna et al., 2024). Rather, the systems in this review–despite being tested only in short-term, often teleoperated interactions–suggest that combining language with intentional touch can shape users’ comfort, trust, and willingness to disclose in ways that linguistic empathy alone cannot. Integrating these strands points toward a longer-term trajectory in which AI caregivers can express support across multiple modalities that more closely resemble those available to human practitioners. However, this raises challenges around consent, appropriate use of touch, and the risk of unsafe reliance on AI systems (Howcroft and Blake, 2025).

### 4.5 Cultural considerations

Culture also shapes attitudes and expectations in HRI (Lim et al., 2021). For example, in a conversational HRI task, Arab and German participants differed in their preferred distance from robots, suggesting that a one-size-fits-all approach to robot proximity may not generalise across cultures (Eresha et al., 2013). Touch behaviours also vary cross-culturally (Sorokowska et al., 2021), which may influence how comfortable users feel with robot-delivered touch. Cultural variation may also shape broader patterns of robot acceptance and adoption, particularly in care contexts where robots may enter intimate space and, in some cases, touch the body. Social robots have seen greater uptake in parts of Asia, particularly Japan, where government policy actively supports care-robot deployment in response to population ageing and workforce pressures (Ministry of Economy, Trade and Industry, and Ministry of Health, Labour and Welfare, 2024). Some studies report that Japanese participants grant greater autonomy to robots and anthropomorphise them more than American participants (Lim et al., 2021). However, while culture is an important factor, it remains unclear how and in which contexts it shapes acceptance of social robots in general, and speech–touch robots in particular.

## 5 Limitations

### 5.1 Design limitations of current speech–touch HRI

#### 5.1.1 Reliance on large, repurposed platforms

Progress in embodied speech–touch systems for affective use is constrained by a shortage of accessible platforms designed for safe, expressive touch. Research on speech–touch integration has largely relied on general-purpose platforms rather than robots purpose-built for affective social touch. Seven of the eleven implementations used widely available research or service robots–PR2 (Arnold and Scheutz, 2018), NAO (Willemse et al., 2017; Willemse and van Erp, 2019), Pepper (Gujran and Jung, 2023; Zhang et al., 2025), and UR3/UR3e (Sawabe et al., 2022; Nieda et al., 2024). Depending on how ‘social robot’ is defined, some platforms–particularly PR2 and UR3/UR3e–may not clearly qualify, given arguments that a social robot should have a form that explicitly signals sociality (Hegel et al., 2009). Their prevalence likely reflects the scarcity of accessible, programmable platforms that support both custom speech interaction and socially supportive touch. This likely encourages researchers either to adapt rigid, general-purpose robots, which are often not designed for close-contact or comforting touch, or to invest substantial time and resources in developing custom-built systems such as Huggable (Logan et al., 2019). Meanwhile, many touch-capable research prototypes and commercial “cuddly” robots are not readily available as programmable platforms for independent researchers, limiting replication and iterative evaluation. Consequently, most existing studies remain proof-of-concept demonstrations, offering limited insight into sustained, affective, naturalistic touch alongside dialogue in everyday settings. At present, the field appears to lack appropriate, accessible platforms for rigorous study.

#### 5.1.2 Hard and mechanical touch

These material constraints may help explain some mixed findings. Of the eight comparative studies, two reported no improvement in psychosocial ratings when combining touch and dialogue (Willemse et al., 2017; Gujran and Jung, 2023). The authors suggested this may be because the robot was perceived as too mechanical and machine-like, and its physical touch lacked the warmth, softness, and lifelike qualities needed for social connection.

#### 5.1.3 Limited tactile sensing and non-adaptive touch

Only three of the eleven implementations used touch sensors; most relied on teleoperated or pre-programmed platforms that delivered touch as a largely fixed, one-way action, with little capacity to treat touch as part of a responsive social exchange. In these studies, robots could not distinguish between different kinds or locations of touch, nor adapt their behaviour accordingly. The absence of sensing is particularly notable because several therapeutic companion robots already employ tactile sensing (e.g., PARO), and studies of these systems indicate that robots which respond contingently to stroking or hugging are associated with higher engagement and calming, comforting socio-emotional effects (Geva et al., 2020).

#### 5.1.4 Restricted autonomy and scripted interactions

Most robots in this review relied on Wizard-of-Oz control or tightly scripted routines, with human operators coordinating speech and touch. While this improves experimental control and safety, it limits scalability and ecological validity, so it is unclear whether comparable effects would occur with autonomous systems in everyday settings. This is especially relevant for LLM-based systems, where latency and generative variability can complicate tightly timed, safety-critical speech–touch coordination.

### 5.2 Future design priorities

The limitations identified suggest several priorities for future robot design in this area (where such work is tractable within sufficiently resourced teams).

#### 5.2.1 Platform size, safety, and autonomy

Reliance on Wizard-of-Oz control and scripted interactions highlights a need for more autonomous systems that can coordinate speech (e.g., via an LLM) and touch without continuous human mediation, especially for scalable care deployments. Yet achieving socially interactive autonomy in social robots can be challenging (Tulli et al., 2019), and safe autonomy is particularly difficult with large, rigid platforms (e.g., PR2, UR3), where small timing or force errors can make close contact unsafe and limit suitability for emotional-support touch. By contrast, small soft robots can offer ‘inherent safety’ through lightweight, compliant bodies, making autonomous tactile behaviours more feasible (Giannaccini et al., 2018). In practice, many companion robots (e.g., PARO) favour user-initiated, responsive touch over robot-initiated contact. This may also support acceptability through perceived safety and approachability.

#### 5.2.2 Embodiment and materials

**FIGURE 5:**
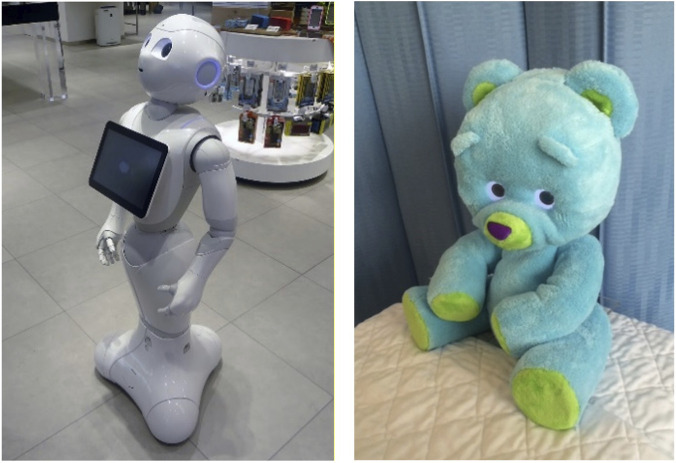
Real photographs of two contrasting platforms. *(Left)* Pepper, a rigid, plastic-bodied humanoid that some participants experienced as mechanical or “cold” (image by Tokumeigakarinoaoshima, via Wikimedia Commons, public domain; CC0 1.0). *(Right)* Huggable, a plush, tactile robot designed for paediatric comfort (photograph © Honey Goodenough, used with permission).

In our review, plush and warm embodiments appeared to support more positive affective responses, whereas hard, cold, or mechanical embodiments could reduce anthropomorphism and evoke uncanniness (Jeong et al., 2015; Willemse et al., 2017; Logan et al., 2019; Block et al., 2023; Gujran and Jung, 2023; Zhang et al., 2025). This contrast is illustrated in Figure 5, where Pepper’s hard embodiment (left) differs markedly from Huggable’s soft, plush form (right). This interpretation is supported by broader companion-robot research, in which older adults preferred familiar, zoomorphic forms with soft, furry shells, whereas hard plastic was often perceived as cold and not “friendly” (Bradwell et al., 2019). Emotionally oriented robots should therefore generally prioritise skin-like warmth, soft/compliant surfaces (where compatible with functional requirements), and gentle forces (e.g., CT-style stroking), and assess perceived “naturalness” through felt experience rather than specifications alone.

### 5.3 Limitations of the evidence and this review

#### 5.3.1 Limitations of the included evidence

The evidence base for this review and the included studies were highly heterogeneous in their designs. With the exception of a single randomised controlled trial (Logan et al., 2019), the included robot systems were primarily exploratory, proof-of-concept laboratory implementations or design prototypes. These studies used non-standardised methods, varied robot types, different interaction tasks, and diverse outcomes. This variation made direct comparison or synthesis difficult, which motivated the narrative, thematic synthesis used in this review. Moreover, none of the included studies explicitly reported participant ethnicity or specifically recruited neurodivergent groups, despite the likely relevance of culture and sensory processing to both verbal and tactile interaction (Neuliep, 2006; Burns et al., 2021), as well as to general acceptance of social robots (Lim et al., 2021). The evidence base is currently too limited and narrow to support meaningful claims about how different populations experience or value speech–touch HRI.

#### 5.3.2 Limitations of this review

This scoping review aims to map the design space of robots that integrate speech with intentional touch for affective use, rather than to synthesise effect sizes or grade evidence across systems. Accordingly, the synthesis should be interpreted as a structured overview of implementation approaches and reported evaluation outcomes, not a comparative assessment of efficacy.

Additionally, because our primary aim was to catalogue robots that combine speech and touch, we mapped evidence to functionally distinct HRI systems rather than listing every evaluation separately. Table 1 therefore consolidates some multi-paper platforms (e.g., Huggable) into a single entry. In some cases, we list the same platform more than once (e.g., NAO) when it was used to implement meaningfully different speech–touch interaction variants. Under this counting approach, we identified 11 implementations. This reflects our aim of mapping the design space: we considered these variants worth separating because they represent distinct ways and results of coordinating speech and touch (e.g., differences in framing or interaction structure). Nevertheless, deciding when a single platform should be treated as one system versus separated into multiple distinct speech–touch HRI systems is not always straightforward, and some consolidation decisions may therefore be less immediately transparent to readers.

Screening was conducted by a single reviewer, which is consistent with the exploratory nature of a scoping review but nevertheless represents a limitation. This may increase the risk of missed records. To enhance methodological rigour, approximately 50% of records were additionally screened using an LLM-assisted workflow to support manual screening, and discrepancies were reviewed. In addition, the completed data charting table (Table 1) and corresponding results summaries were verified by a second reviewer against the original included sources to ensure that study characteristics and findings were accurately represented. However, these procedures did not amount to fully independent duplicate screening or data extraction. Some borderline cases were also difficult to classify, particularly where the distinction between affective/social touch and more functionally oriented contact was not entirely clear (particularly Chen et al., 2014), which may have introduced some subjectivity into eligibility decisions. In addition, some relevant implementations may have been described only in non-indexed or grey sources (e.g., demos, technical reports, or project pages), meaning that some systems may have been inadvertently omitted.

Robot form and material qualities may shape responses to speech–touch interaction. However, our inclusion of representative photographs was limited by copyright and permission constraints. We therefore used GPT-Image 1.5 as part of the process to generate visual representations (Figure 3). Images generated with AI assistance may contain inaccuracies or visual artefacts. Accordingly, these figures are intended only as conceptual visual summaries of broad embodiment and material features, not as exact reproductions of the original platforms or deployable designs.

## 6 Conclusion

To our knowledge, this is the first scoping review to map the nascent research area of robots for social HRI integrating intentional touch with spoken dialogue. Although the evidence base is small, the results suggest that combining speech and touch can, in some contexts, be more effective than speech-only or touch-only HRI. It may make robots seem more caring, empathic, and human-like; strengthen intimacy or attachment; increase willingness to self-disclose; and help people feel calmer or more comfortable (e.g., lower heart rate, more positive affect, and higher pain tolerance).

Speech–touch integration for affective HRI may be most applicable in contexts requiring socio-emotional support (e.g., calming children in hospital). It appears to work best when touch is experienced as comforting and expectation-consistent–warm, soft, compliant rather than cold/rigid. Emotionally oriented robots may therefore benefit from prioritising skin-like warmth and soft, compliant surfaces (where feasible) to avoid cold, rigid, or uncanny sensations.

However, combining touch with dialogue in HRI is fraught with psychological, socio-cultural, and interactional challenges, while also introducing substantial engineering and software complexities. A significant design gap therefore remains. Current research mainly uses repurposed, rigid platforms, and autonomous robots purpose-built for affective speech and touch remain largely absent from the literature.

### 6.1 Protocol and registration

A protocol for this scoping review was registered prospectively with the Open Science Framework on 4 June 2025 (https://doi.org/10.17605/OSF.IO/2PA6J). Deviations from the protocol included: (i) double-screening (for additional rigour) in R with LLM assistance on roughly half of records rather than involving an additional human reviewer, (ii) treating distinct robot implementations as the unit of analysis and, where multiple publications described the same robot, selecting a primary report for inclusion while consulting additional sources for supplementary details; and (iii) refining and narrowing the data items charted to focus on dialogue/touch components, purpose, and key findings.

## Data Availability

The original contributions presented in the study are included in the article/Supplementary Material, further inquiries can be directed to the corresponding author.

## Supplementary material

SUPPLEMENTARY APPENDIX SA Full Boolean search strings and retrieval counts for the five databases searched (IEEE Xplore, MEDLINE/PubMed, ACM Digital Library, Web of Science, Scopus), plus details of manual and grey literature searches. Searches conducted 30 July 2025.
