# Supplementary material for: Speech-touch integration for affective human–robot interaction: a scoping review
Source: Front Robot AI. 2026 May 8;13:1785039. doi: 10.3389/frobt.2026.1785039 (PMC13194039; doi:10.3389/frobt.2026.1785039)
Supplement: Supplementary file 1 [file Supplementaryfile1.docx]

### Multimedia Appendix A. Search queries and number of results for each database

The search focused on four core concepts: *Robot, Verbal Interaction, Tactile Interaction, and Affective/Social Interaction*. These are combined using AND, and group synonyms using OR. Database searches were conducted on 30^th^ July 2025.

| **Database** | **Full Search String** | **Retrieved** |
| --- | --- | --- |
| IEEE Xplore | (  ("robot" OR "robots" OR "embodied agent" OR "social agent" OR "interactive system" OR "companion system" OR "android" OR "conversational agent")  )  AND  (  ("text-to-speech" OR TTS OR "verbal" OR "speech" OR "spoken" OR "dialogue" OR "conversation" OR "linguistic" OR "narrative" OR "utterance")  )  AND  (  ("haptic" OR "tactile" OR "haptic feedback" OR "tactile feedback" OR "hug" OR "embrace" OR "tap" OR "stroking" OR "pressure" OR "actuator")  )  AND  (  ("empathy" OR "social" OR "emotion" OR "affective" OR "compassion" OR "support" OR "caregiver" OR "therapy" OR "rapport" OR "psychology")  ) | 347 |
| MEDLINE (PubMed) | (  Robotics[MeSH Terms] OR  robot*[tiab] OR  embodied agent*[tiab] OR  social agent*[tiab] OR  interactive system*[tiab] OR  companion system*[tiab] OR  android*[tiab] OR  conversational agent*[tiab]  )  AND  (  Speech[MeSH Terms] OR  Language[MeSH Terms] OR  text-to-speech[tiab] OR  TTS[tiab] OR  verbal*[tiab] OR  speech*[tiab] OR  spoken*[tiab] OR  dialog*[tiab] OR  convers*[tiab] OR  linguistic*[tiab] OR  narrative*[tiab] OR  utteranc*[tiab]  )  AND  (  Feedback, Sensory[MeSH Terms] OR  haptic*[tiab] OR  tactile*[tiab] OR  haptic feedback[tiab] OR  tactile feedback[tiab] OR  embrac*[tiab] OR  stroking[tiab] OR  pressure[tiab] OR  contact[tiab] OR  caress*[tiab] OR  vibrotactile[tiab] OR  kinesthetic[tiab] OR  force[tiab] OR  actuator*[tiab]  )  AND  (  Empathy[MeSH Terms] OR  Social Support[MeSH Terms] OR  Patient-Centered Care[MeSH Terms] OR  empath*[tiab] OR  social*[tiab] OR  emotion*[tiab] OR  affect*[tiab] OR  compassion*[tiab] OR  support*[tiab] OR  caregiv*[tiab] OR  therap*[tiab] OR  rapport[tiab] OR  bond*[tiab] OR  psycholog*[tiab] OR  well*[tiab] OR  mental health[tiab] OR  connect*[tiab] OR  interpersonal[tiab] OR  engagement[tiab] OR  companion*[tiab]  ) | 257 |
| ACM Digital Library | ( "robotics" OR robot* OR "embodied agent*" OR "social agent*" OR "interactive system*" OR "companion system*" OR android* OR "conversational agent*" )  AND  ( "text-to-speech" OR TTS OR "speech synthesis" OR "artificial speech" OR verbal* OR speech* OR spoken* OR dialog* OR convers* OR linguistic* OR narrative* OR utteranc* )  AND  ( haptic* OR tactile* OR "haptic feedback" OR "tactile feedback" OR hug* OR embrac* OR tap* OR stroke* OR pressure OR contact OR caress* OR vibrotactile OR kinesthetic OR force OR actuator* )  AND  ( empath* OR social* OR emotion* OR affect* OR compassion* OR support* OR caregiv* OR therap* OR rapport OR bond* OR psycholog* OR well* OR "mental health" OR connect* OR interpersonal OR engagement OR companion* OR "patient-centered care" ) | 103 |
| Web of Science | (  TI=(robot* OR "embodied agent*" OR "social robot*" OR "companion robot*" OR android* OR "conversational agent*")  OR AB=(robot* OR "embodied agent*" OR "social robot*" OR "companion robot*" OR android* OR "conversational agent*")  )  AND  (  TI=("text-to-speech" OR TTS OR speech* OR spoken* OR dialog* OR verbal* OR utteranc*)  OR AB=("text-to-speech" OR TTS OR speech* OR spoken* OR dialog* OR verbal* OR utteranc*)  )  AND  (  TI=(haptic* OR tactile* OR "haptic feedback" OR "tactile feedback" OR hug* OR embrac* OR caress* OR vibrotactile OR touch*)  OR AB=(haptic* OR tactile* OR "haptic feedback" OR "tactile feedback" OR hug* OR embrac* OR caress* OR vibrotactile OR touch*)  )  AND  (  TI=(empath* OR emotion* OR affect* OR compassion* OR therap* OR rapport OR "mental health" OR interpersonal OR engagement)  OR AB=(empath* OR emotion* OR affect* OR compassion* OR therap* OR rapport OR "mental health" OR interpersonal OR engagement)  ) | 103 |
| Scopus | TITLE-ABS-KEY(robot OR robots OR "embodied agent" OR "social agent" OR "interactive system" OR "companion system" OR android OR "conversational agent")  AND  TITLE-ABS-KEY("text to speech" OR TTS OR verbal OR speech OR spoken OR dialog OR conversation OR linguistic OR narrative OR utterance)  AND  TITLE-ABS-KEY(haptic OR tactile OR "haptic feedback" OR "tactile feedback" OR touch OR hug OR embrace OR caress OR vibrotactile OR stroke OR actuator OR kinesthetic)  AND  TITLE-ABS-KEY(empath* OR "emotional support" OR "affective interaction" OR "human robot interaction" OR "therapeutic interaction" OR rapport OR "social touch") | 293 |

**Manual and Grey Literature Searches**In addition to database searches, targeted web searches were conducted to identify known robots potentially exhibiting both verbal and tactile output modalities, tracing peer-reviewed publications where available and using grey literature where necessary. This resulted in sixteen additional results.
